# Bridging the Gap: The Roles of Legacy Leadership and Psychological Safety in Nurse Interns’ Readiness for Practice

**DOI:** 10.1155/jonm/4177127

**Published:** 2026-08-02

**Authors:** Ghada Hamouda, Ebaa Felemban, Maram Banakhar, Mai Yaseen, Khadijah Alshawush, Abdulhafith Alharbi, Ebtsam Aly Abou Hashish

**Affiliations:** ^1^ Public Health Nursing Department, Faculty of Nursing, King Abdulaziz University, Jeddah, Saudi Arabia, kau.edu.sa; ^2^ Nursing Department, Batterjee Medical College, Jeddah, Saudi Arabia, bmc.edu.sa; ^3^ Department of Psychiatric and Mental Health Nursing, College of Nursing, University of Hail, Hail, Saudi Arabia, uoh.edu.sa; ^4^ College of Nursing—Jeddah, King Saud Bin Abdul-Aziz University for Health Sciences, Jeddah, Saudi Arabia, ksau-hs.edu.sa; ^5^ King Abdullah International Medical Research Center, Jeddah, Saudi Arabia, kaimrc.med.sa; ^6^ Ministry of National Guard Health Affairs, Jeddah, Saudi Arabia, ngha.med.sa; ^7^ Faculty of Nursing, Alexandria University, Alexandria, Egypt, alexu.edu.eg

**Keywords:** legacy leadership, nurse internship, nursing education, psychological safety, readiness for practice, Saudi Arabia

## Abstract

**Aim:**

To examine the relationship between legacy leadership and nurse interns’ readiness for clinical practice and to test the moderating role of psychological safety.

**Background:**

The transition from nursing education to clinical practice is a critical period marked by gaps in readiness, limited self‐regulation, and increased patient safety risks. Leadership within clinical settings remains a key modifiable factor shaping this transition. Legacy leadership, grounded in mentorship, ethical modeling, long‐term professional development, and psychological safety have been proposed as facilitators of intern readiness. Their combined role remains underexplored in Saudi Arabia.

**Methods:**

A cross‐sectional predictive correlational design was used. A convenience sample of 178 nurse interns was recruited from four Saudi universities. Data were collected using a self‐administered questionnaire including the Legacy Leadership Scale, Nursing Practice Readiness Scale, and Psychological Safety Scale. Data were analyzed using descriptive statistics, Pearson correlation, an independent samples *t*‐test, and hierarchical regression. All instruments demonstrated strong internal consistency.

**Results:**

Most participants (82.5%) reported high perceptions of legacy leadership (*M* = 107.34, SD = 15.76; 85.9%). Readiness for practice was high (*M* = 117.80, SD = 18.06; 84.1%); self‐regulation was the lowest domain (82.9%). Psychological safety was moderate‐to‐high (*M* = 37.55, SD = 9.47; 76.6%). Legacy leadership showed a significant association with readiness for practice (*β* = 0.757, *R* ^2^ = 0.573, *p* < 0.001). Psychological safety did not significantly moderate this relationship.

**Conclusion:**

Legacy leadership showed the strongest association with readiness for practice and accounted for the largest proportion of explained variance within the regression model. Psychological safety did not function as a moderator. Its role within the leadership readiness relationship may warrant further investigation using alternative explanatory models.

**Implications for Nursing Management:**

Nursing managers should institutionalize structured mentorship development, ethical role modeling, and collaborative empowerment as core legacy leadership competencies within clinical internship frameworks to improve readiness outcomes.

## 1. Introduction

The transition from nursing education to clinical practice is one of the most challenging and consequential phases in professional nursing formation [1, 2]. Nursing graduates entering clinical internships must consolidate knowledge, develop clinical skills, navigate interprofessional environments, and form a professional identity while assuming responsibility for patient care. Nurse interns report uncertainty in decision‐making, lack of confidence, and fear of committing errors that may compromise patient safety [3, 4]. These challenges are associated with increased burnout, turnover intention, and reduced quality of care [2, 5].

In Saudi Arabia, the 1‐year mandatory internship places nurse interns in diverse clinical environments that demand rapid adaptation [6, 7]. Evidence from Saudi settings indicates persistent gaps in readiness for practice. More than half of nurse interns demonstrate only moderate readiness levels, particularly at the beginning of the transition period [8, 9]. These challenges are intensified by broader systemic factors. Rapid academic reforms have been shown to increase psychological distress and reduce self‐efficacy among nursing students, further complicating the transition into clinical practice [10]. These reforms include curriculum restructuring, competency‐based educational standards, revised progression requirements, and an increased emphasis on accreditation and outcome‐based education.

Within this context, leadership experienced during the internship emerges as a critical determinant of readiness development. Legacy leadership represents a leadership approach focused on mentorship, ethical role modeling, and long‐term professional development [11, 12]. Such leadership behaviors have been associated with improved clinical competence, self‐efficacy, and professional readiness among novice nurses [13–16].

Psychological safety has been proposed as a contextual factor influencing how individuals engage in learning and clinical practice. Environments characterized by psychological safety support question‐asking, help‐seeking, and active participation in clinical tasks [17–20]. This suggests that psychological safety may shape the relationship between leadership and readiness outcomes.

Despite these theoretical links, limited research has examined legacy leadership and psychological safety within a single predictive model of nurse intern readiness for practice, particularly in Arab healthcare contexts. The mechanism through which leadership translates into readiness outcomes remains insufficiently understood during the internship transition phase.

This study addresses these gaps by examining legacy leadership as an associated factor of nurse intern readiness for practice and testing the moderating role of psychological safety within the Saudi internship context.

## 2. Background and Literature Review

The following sections synthesize the theoretical and empirical foundations of the study variables. This study is grounded in the conceptualization of three core variables: legacy leadership, readiness for practice, and psychological safety. These constructs collectively provide a framework for understanding how leadership behaviors shape nurse interns’ development during the transition to clinical practice.

### 2.1. Legacy Leadership

Legacy leadership, as conceptualized by Sandstrom and Smith [11], is a leadership philosophy that shifts the focus from individual achievement to the sustained development of others and the long‐term impact of leadership actions. This framework emphasizes intentional mentorship, ethical conduct, and the creation of systems that support future generations of professionals.

The Legacy Leadership Framework comprises five interrelated domains, each contributing to nurse intern development. *Integrity and Ethical Leadership* involves acting with moral consistency, ensuring transparency, and modeling behaviors that build professional trust and accountability. *Mentoring and Empowering Others* focuses on developing team members through structured guidance, delegation, and encouragement of professional ownership. *Collaborative Leadership* promotes teamwork, active listening, and inclusive clinical environments. *Vision and Long-term Impact* reflect the ability to communicate future‐oriented goals and connect present actions to long‐term professional outcomes. *Building Sustainable Systems* emphasizes the development of enduring workflows and the transfer of knowledge across generations of practitioners.

Empirical evidence supports the relevance of these domains. Ethical work climate has been shown to influence organizational commitment, job satisfaction, and professional conduct [21]. Structured mentorship significantly improves self‐confidence, problem‐solving ability, and communication skills among newly graduated nurses [13]. Collaborative leadership has been associated with clinical competence development among Saudi nurse interns [22], while visionary leadership predicts staff satisfaction and organizational commitment across healthcare settings [23].

Evidence from Saudi Arabia further reinforces these patterns. Transformational leadership, which shares key characteristics with legacy leadership, has been shown to improve nursing care quality [24]. Similarly, mentorship, shared vision, and goal alignment have been identified as central supervisory practices among Saudi nurse supervisors [25]. These findings support the contextual relevance of legacy leadership as a framework for understanding intern development in Saudi clinical settings.

### 2.2. Readiness for Practice

Readiness for practice is a multidimensional construct reflecting the integration of knowledge, technical competence, professional attitudes, and interpersonal skills required for safe and effective nursing care [4, 26]. Kim and Shin [26] identified six domains that operationalize readiness for practice.

*Clinical Judgment* refers to the ability to collect and interpret clinical data and make appropriate decisions under uncertainty. *Nursing Performance* represents technical skill proficiency and the delivery of evidence‐based care. *Professional Attitudes* include ethical conduct, accountability, and commitment to patient‐centered care. *Patient-Centeredness* reflects communication, empathy, and advocacy. *Self-egulation* involves emotional control, stress management, and adaptive coping in demanding clinical environments. *Collaborative Interpersonal Relationships* encompass teamwork, communication, and effective help‐seeking behavior [26].

Saudi‐based evidence consistently demonstrates gaps in readiness for practice. Almadani et al. [8] reported that more than half of nurse interns demonstrate only moderate readiness levels, with supervisor support identified as a key determinant. Alruwaili et al. [9] highlighted the influence of training stage and mentorship availability on readiness outcomes. AlThiga et al. [27] found that the quality of supervisor–intern relationships is a primary factor in clinical preparation.

Self‐regulation emerges as the most vulnerable domain. Psychological distress has been shown to reduce self‐efficacy and coping ability among Saudi nursing students [10]. Academic resilience remains moderate and is strongly influenced by environmental support [28]. These findings indicate that leadership behaviors that provide guidance, emotional support, and structured development are critical for strengthening self‐regulatory capacity during an internship.

### 2.3. Psychological Safety in Clinical Learning

Psychological safety is defined as the shared belief that a work environment supports interpersonal risk‐taking, including asking questions, admitting mistakes, and seeking help [17]. It is operationalized through perceptions of freedom from blame, openness to dialog, respect for differences, and recognition of individual contributions.

In clinical education, psychological safety enables key learning behaviors. It supports active participation, encourages questioning, and facilitates skill development. Butler and Mendon [18] demonstrated that psychological safety is essential for clinical engagement and confidence development. Carrillo et al. [19] found that psychological safety is most effectively developed through high‐quality mentorship during clinical training. Dietl et al. [20] reported that psychological safety improves interprofessional communication and patient safety outcomes.

Leadership plays a central role in shaping psychological safety. Transformational leadership has been shown to create trust and openness within teams [29]. Humble leadership behaviors, including openness and approachability, have also been linked to increased psychological safety [30]. These findings suggest that leadership behaviors may not only influence readiness directly but may also shape the psychological conditions under which learning occurs.

### 2.4. Conceptual Framework

Based on these conceptual foundations, the study adopts a framework integrating legacy leadership, readiness for practice, and psychological safety. Legacy leadership is positioned as the independent variable, readiness for practice as the dependent variable, and psychological safety as a moderating variable. The framework is grounded in Bandura’s [31] Social Learning Theory, which explains how individuals learn through observation, modeling, and interaction with their environment.

**FIGURE 1:**
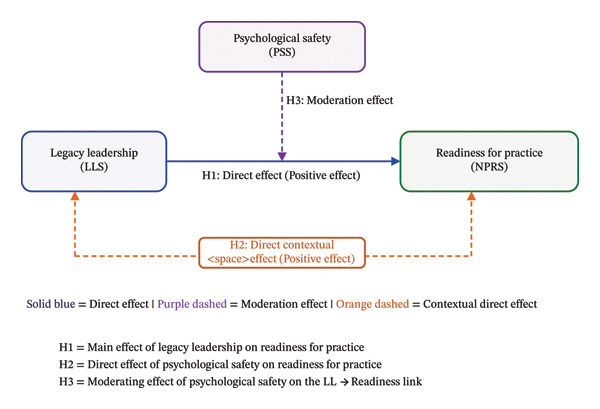
Proposed conceptual framework.

Within this framework, legacy leadership behaviors provide the modeling and guidance necessary for skill acquisition and professional development. Psychological safety represents the contextual condition that may influence how effectively these leadership behaviors translate into readiness outcomes. The framework is supported by empirical evidence demonstrating that environmental support, mentorship quality, and leadership behaviors significantly influence intern development [8–10, 28] (Figure 1). This framework assumes that leadership behaviors influence readiness both directly and through contextual learning conditions.

### 2.5. Study Hypotheses

H1. Legacy leadership is positively associated with nurse interns’ readiness for practice.
H2. Psychological safety is positively associated with nurse interns’ readiness for practice.
H3. Psychological safety moderates the relationship between legacy leadership and readiness for practice, such that the relationship is stronger at higher levels of psychological safety.

### 2.6. Research Gap

Despite these converging theoretical links, several empirical gaps remain. First, legacy leadership has not been examined as a distinct and operationalized construct within nursing internship contexts. Existing research in nursing leadership focuses primarily on transformational, authentic, or servant leadership styles [23, 24], leaving the unique contribution of legacy leadership insufficiently explored. Second, no study has tested legacy leadership and psychological safety within a single predictive model of nurse intern readiness for practice. The interaction between leadership behaviors and contextual conditions shaping readiness outcomes remains underexplored. Third, Saudi Arabia and the broader Arab region remain underrepresented in the global literature on readiness for practice, despite documented regional gaps in nurse intern readiness, mentorship support, and transition‐to‐practice outcomes across Saudi and Arab healthcare settings [8, 9] and ongoing healthcare transformation initiatives, including healthcare privatization, workforce nationalization, digital transformation, and quality improvement initiatives under Saudi Vision 2030. This study addresses these gaps through an integrated empirical examination of leadership, context, and readiness within the Saudi nursing internship setting.

### 2.7. Significance of the Study

This study contributes at multiple levels. At the theoretical level, it provides an empirical test of legacy leadership association with nurse intern readiness for practice within a moderated regression framework [4, 32] and introduces legacy leadership as a distinct construct within nursing research. At the clinical level, identifying leadership behaviors associated with readiness development has direct implications for patient safety, given the documented prevalence of moderate readiness among nurse interns in Saudi settings [8, 9] and the established link between leadership quality and patient safety culture [20]. At the educational level, the study extends existing evidence demonstrating that structured developmental support enhances readiness, self‐efficacy, and well‐being among nursing students and interns [10, 15, 16, 28]. At the policy level, the findings support Vision 2030 priorities aimed at strengthening workforce capability, improving healthcare quality, expanding nursing leadership capacity, and promoting sustainable professional development.

### 2.8. Aim of the Study

Accordingly, this study aims to examine the relationship between legacy leadership and nurse interns’ readiness for clinical practice and to test the moderating role of psychological safety within the Saudi nursing internship context.

## 3. Methods

### 3.1. Study Design

A cross‐sectional correlational design was employed to examine the relationships among legacy leadership, psychological safety, and nurse interns’ readiness for practice within a real‐world clinical context. The study was conducted and reported in accordance with the Strengthening the Reporting of Observational Studies in Epidemiology (STROBE) guidelines for cross‐sectional studies.

### 3.2. Setting, Sample, and Recruitment

A convenience sampling technique was used to recruit nurse interns completing the mandatory 1‐year clinical internship at healthcare institutions affiliated with four Saudi universities. Inclusion criteria required active enrollment in the internship program during the data collection period and provision of electronic informed consent. Exclusion criteria included non‐nursing interns and surveys with more than 20% missing responses on any scale. The final sample comprised 178 nurse interns.

### 3.3. Sample Size and Power Analysis

A priori power analysis was conducted using G × Power version 3.1 for hierarchical multiple regression. To detect a medium effect size (*f* ^2^ = 0.15) with *α* = 0.05, power = 0.80, and three predictors, the minimum required sample size was *n* = 77. For moderation analysis, assuming a small‐to‐medium interaction effect (*f* ^2^ = 0.08), the required sample increased to *n* = 122. The achieved sample size (*N* = 178) exceeded both thresholds, indicating adequate statistical power for all planned analyses.

### 3.4. Instruments

Data were collected using a structured self‐administered questionnaire consisting of four sections.

The first section included a researcher‐developed sociodemographic form capturing age, gender, daily working hours, type of healthcare organization, ward specialty, training stage, marital status, city, and university affiliation.

The second section comprised the Legacy Leadership Scale (LLS), a researcher‐developed instrument (2025) measuring nurse interns’ perceptions of leadership behaviors across five domains: integrity and ethical leadership, mentoring and empowering others, collaborative leadership, vision and long‐term impact, and building sustainable systems. Each domain includes five items, yielding a total of 25 items. Responses are rated on a five‐point Likert scale (1 = strongly disagree to 5 = strongly agree), with total scores ranging from 25 to 125. Scores were interpreted as follows: ≥ 95 indicates high perception of legacy leadership, 75–94 moderate, and < 75 low.

The third section included the nursing practice readiness scale (NPRS) developed by Kim and Shin [26]. The scale consists of 35 items distributed across six domains: clinical judgment (6 items), nursing performance (9 items), professional attitudes (5 items), patient‐centeredness (5 items), self‐regulation (5 items), and collaborative interpersonal relationships (5 items). Items are rated on a four‐point Likert scale (1 = strongly disagree to 4 = strongly agree), with total scores ranging from 35 to 140. Higher scores indicate greater readiness for clinical practice.

The fourth section included Edmondson’s [17] Psychological Safety Scale (PSS), which consists of seven items measuring perceived interpersonal safety within the clinical environment. Items are rated on a seven‐point Likert scale (1 = strongly disagree to 7 = strongly agree), with total scores ranging from 7 to 49. Items 1, 3, and 5 were negatively worded statements and were reverse‐coded before total score calculation so that higher scores consistently reflected greater psychological safety. Higher scores reflect greater perceived psychological safety. The four elements depicted within the psychological safety construct in the conceptual framework (Figure 1)—speaking up freely, asking for help, admitting mistakes, and engaging in interpersonal risk‐taking—are illustrative thematic representations of the construct rather than the individual scale items, which comprise the seven items of Edmondson’s instrument.

### 3.5. Translation and Instrument Equivalence

A forward–backward translation procedure was applied to ensure linguistic and conceptual equivalence of the Arabic version. Two independent bilingual nursing scholars translated the instruments into Arabic. Discrepancies were resolved through consensus with a third bilingual expert. An independent translator then performed back‐translation into English. Semantic, idiomatic, and conceptual equivalence were confirmed through expert review. Cultural appropriateness was verified by two Saudi nursing faculty members.

### 3.6. Validity and Reliability

Content validity of the LLS was established through expert panel review by five nursing administration specialists. Each item was evaluated for clarity, relevance, and representativeness using a four‐point scale. The Item‐level Content Validity Index ranged from 0.80 to 1.00, and the Scale‐level Content Validity Index (S‐CVI/Ave) was 0.92, exceeding recommended thresholds. Face validity was confirmed through pilot testing with 18 nurse interns. Internal consistency was excellent (Cronbach’s *α* = 0.981), indicating strong item coherence, although the high alpha suggests potential item redundancy that warrants further investigation.

Construct validity of the LLS was explored using exploratory factor analysis on the subset of participants with complete item‐level data (*n* = 89). Sampling adequacy was supported by a Kaiser–Meyer–Olkin value of 0.898, and Bartlett’s test of sphericity was significant, χ^2^(300) = 2596.31, *p* < 0.001. Eigenvalue examination revealed a dominant first factor explaining 65.12% of the variance, with three factors exceeding an eigenvalue of 1.00. Although only three factors met the Kaiser criterion, a theory‐driven five‐factor solution was examined because it reflected the conceptual structure of the scale. This five‐factor solution explained 80.70% of the total variance. Given the limited item‐level sample size, these findings should be interpreted as preliminary exploratory evidence rather than full construct validation. Detailed psychometric results are presented in Supporting Tables S2–S5. All three tools demonstrated excellent internal consistency, with Cronbach’s *α* = 0.981 for the LLS, 0.984 for the NPRS, and 0.903 for PSS.

### 3.7. Ethical Considerations

Ethical approval was obtained from the Nursing Research Ethical Committee, King Abdulaziz University (NREC Ref No. 2F.112). Electronic informed consent was obtained from all participants. Participation was voluntary, and anonymity was ensured by not collecting identifying information. Data were stored securely in password‐protected files. The study adhered to the principles of the Declaration of Helsinki.

### 3.8. Statistical Analysis

Data were analyzed using IBM SPSS Statistics version 22 (IBM Corp., Armonk, NY, USA). Descriptive statistics were used to summarize participant characteristics and study variables. Internal consistency was evaluated using Cronbach’s alpha coefficients. Pearson’s correlation analysis was performed to examine relationships among the study variables. Independent‐samples *t*‐tests were conducted to compare study variables by gender.

Hierarchical moderated multiple regression analysis was performed in three sequential models. Model one examined the association between legacy leadership and readiness for practice. Model 2 added psychological safety as a main‐effect predictor. Model 3 introduced the interaction term (legacy leadership × psychological safety) to test the hypothesized moderating effect of psychological safety. A sensitivity analysis adjusted for gender, age, internship stage, and university affiliation. Continuous predictor variables were standardized before creating the interaction term to reduce potential multicollinearity. Regression results are presented as unstandardized coefficients (B), standardized coefficients (*β*), standard errors (SE), *t*‐values, 95% confidence intervals (CIs), coefficients of determination (*R* ^2^), changes in explained variance (Δ*R* ^2^), and F‐statistics. Statistical significance was set at *p* < 0.05 (two‐tailed).

To provide additional descriptive information, participants were categorized into high‐ and low‐readiness groups using the sample median score of the Readiness for Practice Scale. Baseline characteristics were compared between the two groups using Pearson’s *χ* ^2^ test or Fisher’s exact test, as appropriate. This categorization was performed solely for descriptive purposes; all regression analyses retained readiness for practice as a continuous variable.

## 4. Results

### 4.1. Sociodemographic Characteristics

Table 1 presents the sociodemographic characteristics of the participants. The sample was predominantly female (59.6%) and single (95.5%). The mean age was 23.41 ± 1.12 years, with the majority aged 21–23 years (83.1%). Hail University contributed the largest proportion of participants (*n* = 106; 59.6%), followed by Batterjee Medical College (*n* = 40; 22.5%), Jouf University (*n* = 16; 9.0%), and King Abdulaziz University (*n* = 16; 9.0%). Over half of the sample (52.8%) were in the final stage of the internship (9–12 months), indicating substantial clinical exposure. Emergency (27.0%) and surgical (20.2%) placements were the most common. Most participants were trained in governmental healthcare settings (84.3%), reflecting the predominance of government‐funded healthcare services within Saudi Arabia, where most internship placements occur in Ministry of Health, university‐affiliated, military, or National Guard hospitals.

**TABLE 1:** Sociodemographic characteristics of participating nurse interns (*N* = 178).

Variable	Category	n	%
Gender	Female	106	59.6%
	Male	72	40.4%

Age	21–23 years	148	83.1%
	24–26 years	26	14.6%
	> 26 years	4	2.2%

Marital status	Single	170	95.5%
	Married	6	3.4%
	Divorced	2	1.1%

University	Hail University	106	59.6%
	Batterjee Medical College	40	22.5%
	Jouf University	16	9.0%
	King Abdulaziz University	16	9.0%

Training stage	Beginning (1–4 months)	74	41.6%
	Middle (5–8 months)	10	5.6%
	End (9–12 months)	94	52.8%

Ward specialty	Emergency	48	27.0%
	Surgical	36	20.2%
	ICU/critical care	34	19.1%
	Medical	32	18.0%
	Pediatric	14	7.9%
	OB/GYN	14	7.9%

Working hours/day	8 h	138	77.5%
	12 h	34	19.1%
	Other	6	3.4%

Healthcare setting	Governmental	150	84.3%
	Military	14	7.9%
	Private	8	4.5%
	Other	6	3.4%

### 4.2. Descriptive Statistics of Studied Variables

Table 2 presents the descriptive statistics of the study variables and their domains, which indicate consistently high scores across all domains, with relatively lower values observed for psychological safety compared to legacy leadership and readiness for practice. The overall mean LLS score was 107.34 ± 15.76, representing 85.9% of the maximum possible score. The majority of participants (82.5%) reported high levels of legacy leadership perception, while 17.5% (*n* = 31) reported moderate levels, and none fell within the low category.

**TABLE 2:** Descriptive statistics of study variables and domains.

Scale/domain	M	SD	% of max
Legacy Leadership Scale (total)	**107.34**	**15.76**	**85.9%**
Integrity and Ethical Leadership	21.61	3.09	86.4%
Mentoring and Empowering Others	21.36	3.19	85.4%
Collaborative Leadership	21.44	3.28	85.7%
Vision and Long‐Term Impact	21.25	3.58	85.0%
Building Sustainable Systems	21.34	3.44	85.4%
Readiness for Practice Scale (total)	**117.8**	**18.06**	**84.1%**
Clinical Judgment	20.29	3.34	84.5%
Nursing Performance	30.14	4.91	83.7%
Professional Attitudes	16.92	2.59	84.6%
Patient‐Centeredness	16.96	2.75	84.8%
Self‐Regulation	16.58	2.88	82.9%
Collaborative Interpersonal Relationships	16.68	2.76	83.4%
Psychological Safety Scale (total)	**37.55**	**9.47**	**76.6%**

*Note:* M = mean. Bold values reflect the overall mean.

Abbreviation: SD = standard deviation.

Among the domains, Integrity and Ethical Leadership demonstrated the highest mean score (*M* = 21.61, SD = 3.09; 86.4%), whereas Vision and Long‐term Impact showed the lowest (*M* = 21.25, SD = 3.58; 85.0%), although differences across domains were minimal.

The overall mean NPRS score was 117.80 ± 18.06 (range = 70–140), representing 84.1% of the maximum possible score and a mean item score of 3.37/4.00. Patient‐Centeredness demonstrated the highest domain score (*M* = 16.96, SD = 2.75; 84.8%), whereas Self‐Regulation was the lowest (*M* = 16.58, SD = 2.88; 82.9%). These findings indicate generally high readiness levels, with comparatively lower performance in self‐regulatory competencies.

The mean PSS score was 37.55 ± 9.47 (range = 7–49), corresponding to 76.6% of the maximum possible score and a mean item score of 5.37/7, indicating moderate‐to‐high perceived psychological safety. See Supporting Table S1 for PSS item scores.

To provide additional descriptive insight into the sample, participants were categorized into low‐ and high‐readiness groups using the median score of the Readiness for Practice Scale. Comparisons of baseline characteristics are presented in Table 3. This categorization was used solely for descriptive purposes, whereas all inferential analyses retained readiness for practice as a continuous variable.

**TABLE 3:** Comparison of participant characteristics according to readiness for practice level.

Variable	Category	Low readiness *n* (%)	High readiness *n* (%)	Test *p*
Gender	Female	52 (59.1)	54 (60.0)	*χ* ^2^ 1.000
	Male	36 (40.9)	36 (40.0)	

Age	21–23 years	74 (84.1)	74 (82.2)	*χ* ^2^ 0.894
	≥ 24 years	14 (15.9)	16 (17.8)	

Marital status	Single	86 (97.7)	84 (93.3)	Fisher’s exact 0.278
	Married/divorced	2 (2.3)	6 (6.7)	

University	Hail University	50 (56.8)	56 (62.2)	*χ* ^2^ 0.082
	Batterjee Medical College	18 (20.5)	20 (22.2)	
	Jouf University	14 (15.9)	4 (4.4)	
	King Abdulaziz University	6 (6.8)	8 (8.9)	
	Not specified	0 (0.0)	2 (2.2)	

Training stage	Beginning (1–4 months)	42 (47.7)	32 (35.6)	*χ* ^2^ 0.247
	Middle (5–8 months)	4 (4.5)	6 (6.7)	
	End (9–12 months)	42 (47.7)	52 (57.8)	

Ward specialty	Emergency	24 (27.3)	24 (26.7)	*χ* ^2^ 0.001
	Surgical	12 (13.6)	24 (26.7)	
	ICU (critical)	20 (22.7)	14 (15.6)	
	Medical	24 (27.3)	8 (8.9)	
	Pediatric	6 (6.8)	8 (8.9)	
	OB/GYN	2 (2.3)	12 (13.3)	

Working hours	8 h	68 (77.3)	70 (77.8)	*χ* ^2^ 0.673
	12 h	18 (20.5)	16 (17.8)	
	Other	2 (2.3)	4 (4.4)	

Healthcare setting	Governmental	82 (93.2)	68 (75.6)	*χ* ^2^ 0.008
	Military	4 (4.5)	10 (11.1)	
	Private	2 (2.3)	6 (6.7)	
	Other	0 (0.0)	6 (6.7)	

*Note:* Participants were categorized using the sample median of the Readiness for Practice Scal (Median = 117.0). Pearson’s *χ* ^2^ test was used unless expected cell counts were < 5, in which case Fisher’s exact test was applied.

### 4.3. Bivariate Correlations

Table 4 illustrates the Pearson correlation coefficients among the study variables. A strong, positive, and statistically significant correlation was found between legacy leadership and readiness for practice (*r* = 0.757, *p* < 0.001), supporting Hypothesis H1. Legacy leadership showed a small, non‐significant positive correlation with psychological safety (*r* = 0.112, *p* = 0.206), while readiness for practice also demonstrated a small, non‐significant correlation with psychological safety (*r* = 0.070, *p* = 0.427).

**TABLE 4:** Pearson correlation matrix: legacy leadership, readiness for practice, and psychological safety.

Variable	1	2	3
1. Legacy leadership	—		
2. Readiness for practice	0.757^∗∗∗^	—	
3. Psychological safety	0.112	0.070	—

*Note:* Pearson correlation coefficient values are presented.

^∗∗∗^ *p* < 0.001.

These weak and non‐significant associations between psychological safety and the other variables provide important context for interpreting the subsequent moderation analysis, particularly the absence of a significant interaction effect.

### 4.4. Hierarchical Regression Analysis

Hierarchical moderated regression analysis was conducted to examine the relationships among legacy leadership, psychological safety, and readiness for practice (Table 5). Model 1 demonstrated that legacy leadership was a significant positive predictor of readiness for practice (*B* = 0.76, *β* = 0.757, *t* = 13.10, *p* < 0.001), explaining 57.3% of the variance in readiness for practice (*R* ^2^ = 0.573, adjusted *R* ^2^ = 0.569), thereby supporting H1.

**TABLE 5:** Hierarchical moderated regression analysis predicting readiness for practice.

Predictor	B	SE	*β*	*t*	*p*	95% CI [LL, UL]
Model 1. Legacy Leadership						
Legacy leadership	0.76	0.06	0.757	13.10	< 0.001^∗^	[0.64, 0.87]
*R* ^2^ = 0.573; Adjusted *R* ^2^ = 0.569; *F*(1, 128) = 171.51, *p* < 0.001						
Model 2. Adding Psychological Safety						
Legacy leadership	0.76	0.06	0.758	12.99	< 0.001^∗^	[0.64, 0.87]
Psychological safety	−0.01	0.06	−0.014	−0.25	0.806	[−0.13, 0.10]
*R* ^2^ = 0.573; Adjusted *R* ^2^ = 0.566; Δ*R* ^2^ < 0.001; Δ*F*(1, 127) = 0.06, *p* = 0.806						
Model 3. Testing the Moderating Effect						
Legacy leadership	0.76	0.06	0.758	12.92	< 0.001^∗^	[0.64, 0.87]
Psychological safety	−0.01	0.06	−0.007	−0.11	0.916	[−0.13, 0.12]
Legacy leadership × Psychological safety	−0.02	0.07	−0.021	−0.32	0.751	[−0.15, 0.11]
*R* ^2^ = 0.573; Adjusted *R* ^2^ = 0.563; Δ*R* ^2^ = 0.001; Δ*F*(1, 126) = 0.10; *p* = 0.751						

*Note:* B = unstandardized coefficient; *β* = standardized coefficient.

Abbreviations: CI = confidence interval, LL/UL = lower/upper limit, SE = standard error.

^∗^ *p* < 0.001.

In Model 2, psychological safety was added to the regression model. Legacy leadership remained a significant positive predictor (*B* = 0.76, *β* = 0.758, *p* < 0.001), whereas psychological safety was not significantly associated with readiness for practice (*B* = −0.01, *β* = −0.014, *p* = 0.806). The addition of psychological safety did not improve the model (Δ*R* ^2^ < 0.001), indicating that H2 was not supported.

Model 3 examined the moderating effect of psychological safety by introducing the interaction term between legacy leadership and psychological safety. The interaction was not statistically significant (*B* = −0.02, *β* = −0.021, *p* = 0.751), and the explained variance remained essentially unchanged (Δ*R* ^2^ = 0.001). These findings indicate that psychological safety did not moderate the relationship between legacy leadership and readiness for practice; therefore, H3 was not supported.

An additional regression analysis was performed after adjusting for gender, age, internship stage, and university affiliation (Table 6). The adjusted model explained 70.6% of the variance in readiness for practice (*R* ^2^ = 0.706, adjusted *R* ^2^ = 0.679, Δ*R* ^2^ = 0.133, ΔF(8, 118) = 6.67, *p* < 0.001). Consistent with the primary analysis, legacy leadership remained the strongest positive predictor of readiness for practice (*B* = 0.87, *β* = 0.865, *p* < 0.001). Psychological safety remained non‐significant (*B* = −0.05, *β* = −0.054, *p* = 0.356), and the interaction between legacy leadership and psychological safety also remained non‐significant (*B* = −0.03, *β* = −0.027, *p* = 0.666), confirming that the moderation effect was not supported after adjustment for sociodemographic characteristics.

**TABLE 6:** Adjusted multiple regression predicting readiness for practice after controlling for sociodemographic characteristics.

Predictor	B	SE	*β*	t	*p*	95% CI [LL, UL]
Model 4. Adjusted for Sociodemographic Characteristics						
Male (vs. female)	−0.10	0.11	−0.047	−0.87	0.384	[−0.31, 0.12]
Age 24–26 years (vs. 21–23 years)	−0.61	0.15	−0.228	−4.10	< 0.001	[−0.90, −0.31]
Age > 26 years (vs. 21–23 years)	−0.26	0.47	−0.032	−0.56	0.575	[−1.19, 0.66]
Middle internship stage (vs. beginning)	−1.16	0.23	−0.279	−5.02	< 0.001	[−1.61, −0.70]
End internship stage (vs. beginning)	0.09	0.16	0.046	0.58	0.564	[−0.22, 0.41]
Batterjee Medical College (vs. Hail University)	0.55	0.18	0.239	3.06	0.003	[0.20, 0.91]
Jouf University (vs. Hail University)	1.19	0.47	0.147	2.55	0.012	[0.27, 2.12]
King Abdulaziz University (vs. Hail University)	0.17	0.24	0.046	0.72	0.475	[−0.31, 0.65]
Legacy leadership	0.87	0.06	0.865	15.38	< 0.001	[0.75, 0.98]
Psychological safety	−0.05	0.06	−0.054	−0.93	0.356	[−0.17, 0.06]
Legacy leadership × psychological safety	−0.03	0.07	−0.027	−0.43	0.666	[−0.16, 0.10]
Model summary: *R* ^2^ = 0.706; adjusted *R* ^2^ = 0.679; Δ*R* ^2^ = 0.133; ΔF(8, 118) = 6.67, *p* < 0.001						

*Note:* B = unstandardized coefficient; *β* = standardized coefficient.

Abbreviations: CI = confidence interval; LL/UL = lower/upper limit; SE = standard error.

^∗^ *p* < 0.001.

Among the covariates, interns aged 24–26 years reported significantly lower readiness for practice than those aged 21–23 years (*B* = −0.61, *β* = −0.228, *p* < 0.001). Similarly, interns in the middle stage of the internship demonstrated significantly lower readiness than those at the beginning of the internship (*B* = −1.16, *β* = −0.279, *p* < 0.001). Compared with interns from Hail University, those from Batterjee Medical College (*B* = 0.55, *β* = 0.239, *p* = 0.003) and Jouf University (*B* = 1.19, *β* = 0.147, *p* = 0.012) reported significantly higher readiness for practice. Gender, interns older than 26 years, end‐stage internship status, and King Abdulaziz University affiliation were not significantly associated with readiness for practice.

## 5. Discussion

To our knowledge, this study is the first to examine the relationships among legacy leadership, psychological safety, and readiness for practice among nurse interns. By introducing legacy leadership into the nursing internship context, the study extends current leadership literature and provides new evidence of its association with readiness for practice within the Saudi healthcare setting. The findings are interpreted in relation to Social Learning Theory, existing international and Saudi evidence, and their implications for nursing leadership, internship education, and workforce development.

### 5.1. Legacy Leadership Perceptions: High Endorsement and Domain‐Level Insights

More than four‐fifths of nurse interns perceived high levels of legacy leadership, suggesting that the participating clinical settings foster leadership environments characterized by mentoring, ethical practice, collaboration, long‐term vision, and system stewardship. These findings imply that legacy leadership behaviors are consistently experienced during internship training and may provide an important foundation for interns’ professional development. Similar findings have been reported by Lagura et al. [25], who identified high levels of transformational leadership among Saudi nurse supervisors, particularly in mentoring and empowerment. Likewise, Alilyyani et al. [22] demonstrated that leadership exposure positively influenced clinical competence among Saudi nurse interns, while Ystaas et al. [23] concluded that relationship‐centered leadership consistently promotes favorable professional outcomes across healthcare settings.

Integrity and ethical leadership emerged as the highest‐rated legacy leadership domain, emphasizing the importance of leaders’ day‐to‐day conduct during internship training. Because nurse interns interact primarily with frontline supervisors rather than senior administrators, they are more likely to evaluate leadership through observable behaviors such as fairness, integrity, respect, and ethical decision‐making than through strategic or organizational vision. This interpretation is consistent with Social Learning Theory [31], which proposes that professional behaviors are acquired through observing credible role models. Within healthcare settings where ethical practice and professional values are strongly emphasized, these leadership behaviors are likely to shape interns’ professional identity, confidence, and standards of practice. Similar observations were reported by Boshra et al. [24], who found that transformational leadership improved nursing care quality, whereas Abou Hashish [21] demonstrated that an ethical work climate enhanced organizational commitment, job satisfaction, and professional conduct among nurses. Together, these findings highlight ethical leadership as a key element in developing competent and professionally prepared nurses.

By contrast, vision and long‐term impact received comparatively lower ratings. Rather than reflecting weaknesses in leadership practice, this finding may indicate that interns naturally prioritize immediate clinical responsibilities, skill acquisition, and safe patient care during the early stages of professional development. This interpretation is consistent with Benner’s model of skill acquisition, which proposes that novice practitioners initially focus on mastering immediate clinical tasks before developing broader professional and organizational perspectives [4]. Similarly, AlThiga et al. [27] and Almadani et al. [8] emphasized that internship experiences are primarily directed toward building clinical competence and confidence, with greater appreciation of organizational and leadership roles developing as professional experience increases. As interns gain clinical experience and assume greater responsibility, they may become more aware of leaders’ contributions to organizational development, quality improvement, and long‐term workforce sustainability. This progression suggests that some dimensions of legacy leadership may become increasingly salient as nurses mature professionally and assume broader organizational responsibilities. Future longitudinal studies should examine how perceptions of different legacy leadership dimensions evolve across the early stages of nurses’ careers.

### 5.2. Readiness for Practice: Broad Competence and the Self‐Regulation Priority

The high overall readiness for practice scores suggest that Saudi nursing internship programs effectively prepare interns for the transition to professional practice by developing competence across multiple clinical and professional domains. These findings reflect the structured nature of internship training within Saudi healthcare institutions, where supervised clinical experiences, competency‐based learning, and progressive responsibility enable interns to translate theoretical knowledge into safe and effective patient care. Similar findings have been reported by AlThiga et al. [27], who found that both nurse interns and faculty members perceived internship programs as effective in preparing graduates for clinical practice. Likewise, Almadani et al. [8] reported generally high levels of readiness among interns completing structured internship training. The particularly high ratings for Patient‐Centeredness and Professional Attitudes further emphasize the strong focus placed on compassionate care, professional values, and ethical responsibility throughout undergraduate nursing education and internship programs.

The comparison between interns classified as having high and low readiness for practice provides additional insight into factors associated with successful transition to practice. Interns with high readiness were more likely to be younger, in the early stage of their internship, and enrolled at specific universities than those with lower readiness, whereas marital status was not associated with readiness (Table 3). These findings suggest that readiness for practice is shaped primarily by educational experiences and the clinical learning environment rather than by personal demographic characteristics. Differences observed across participating universities may reflect variation in clinical supervision, learning opportunities, organizational culture, or internship implementation, although these organizational characteristics were not examined directly in the present study. Future multicenter studies should investigate these contextual factors to better understand how organizational environments contribute to interns’ readiness for professional practice.

Despite these encouraging findings, self‐regulation emerged as the lowest‐rated readiness domain, highlighting an important developmental priority during the transition‐to‐practice period. Self‐regulation encompasses emotional control, stress management, clinical confidence, and the ability to adapt effectively under pressure. These competencies are often challenged as interns move beyond the initial orientation period and begin assuming greater clinical responsibility while continuing to develop confidence and independent clinical judgment. This interpretation is consistent with Alsayed et al. [10], who reported increased psychological distress and reduced self‐efficacy among Saudi nursing students during periods of educational transition. Similarly, Abou Hashish et al. [28] found that academic resilience was only moderate and was strongly influenced by environmental support. Comparable findings have been reported internationally, with Güner [3] and Zhao et al. [2] identifying emotional regulation and adaptive coping as persistent challenges among newly graduated nurses during their transition into professional practice.

The prominence of self‐regulation also has important implications for nursing leadership. Evidence consistently demonstrates that supportive leadership and structured mentorship strengthen novice nurses’ confidence, coping ability, and professional development. Gularte‐Rinaldo et al. [13] found that sustained mentorship improved self‐confidence and resilience among novice nurses, while Jung and Kim [14] showed that reflective mentorship facilitated professional identity formation and eased the transition into practice. Likewise, Khalil and Abou Hashish [7] demonstrated that reflective practice enhanced critical thinking, an essential component of effective self‐regulation. Together, these findings suggest that leadership approaches emphasizing mentoring, constructive feedback, empowerment, and reflective learning may be particularly valuable for strengthening interns’ self‐regulatory capacity and promoting a successful transition to professional nursing practice.

### 5.3. Legacy Leadership and Readiness for Practice

The strong positive association between legacy leadership and readiness for practice represents the principal finding of this study and provides the first empirical evidence supporting legacy leadership as a meaningful framework for nursing internship education. Legacy leadership explained a substantial proportion of the variance in readiness for practice, and this relationship remained stable across the hierarchical regression models. Moreover, the association remained statistically significant after adjustment for gender, age, internship stage, and university affiliation (Table 6), indicating that its influence was independent of measured participant characteristics. The strong correlation between legacy leadership and readiness for practice further reinforces this finding and supports the robustness of the proposed framework.

Several complementary mechanisms may explain this relationship. Consistent with Social Learning Theory [31], leaders who consistently demonstrate professional competence, ethical conduct, and effective clinical decision‐making provide observable role models that interns can emulate in their own practice. In addition, mentoring and supportive supervisory relationships foster professional confidence, facilitate identity formation, and help interns navigate the uncertainty associated with the transition from student to registered nurse [13, 14]. Leadership also promotes knowledge sharing, coaching, and experiential learning, enabling interns to translate theoretical knowledge into competent clinical practice while narrowing the theory–practice gap that remains a persistent challenge within Saudi nursing education [8, 9].

These interpretations are supported by previous leadership research. Boshra et al. [24] demonstrated that transformational leadership improves nursing care quality, while Lagura et al. [25] identified mentoring, empowerment, and shared goal setting as core supervisory practices that enhance professional development. Likewise, Abou Hashish et al. [33] showed that leadership support facilitates evidence‐based practice implementation, suggesting that leadership continues to shape nurses’ professional growth well beyond the internship period. Together, these findings indicate that legacy leadership integrates ethical role modeling, mentoring, knowledge transfer, and long‐term professional development into a coherent leadership approach. Developing these leadership behaviors among nurse preceptors and clinical supervisors may therefore strengthen interns’ readiness for practice and facilitate a more confident transition into the nursing workforce.

### 5.4. Psychological Safety: Non‐significant Moderation and Interpretive Implications

Contrary to the proposed hypothesis, psychological safety did not moderate the relationship between legacy leadership and readiness for practice. Furthermore, psychological safety was not independently associated with readiness for practice in either the primary or adjusted regression analyses. Although these findings did not support the hypothesized moderating role, they provide useful insight into how leadership influences interns’ transition into professional practice.

One possible explanation is the relatively limited variability in psychological safety scores. Most interns reported moderate‐to‐high levels of psychological safety, suggesting that the participating internship settings generally provided supportive learning environments. Consequently, psychological safety may have reached a functional threshold across these settings, reducing its ability to distinguish differences in readiness for practice. In structured internship programs where orientation, supervision, and preceptorship are routinely provided, supportive interpersonal environments may be expected rather than exceptional.

The findings also suggest that the influence of legacy leadership may be sufficiently strong to operate regardless of modest differences in perceived psychological safety. The persistence of the leadership effect across both the primary and adjusted regression analyses supports this interpretation. Similarly, Carrillo et al. [19] reported that psychological safety is most effective when embedded within mentoring relationships, suggesting that it may function as an integral component of effective leadership rather than as an independent moderator.

Another possible interpretation is that psychological safety operates through mechanisms other than moderation. Previous research has shown that leadership behaviors contribute to psychologically safe work environments [29, 30], raising the possibility that psychological safety may represent an intermediate process linking leadership with professional outcomes. However, because mediation was not examined in the present study, this interpretation remains speculative and should be evaluated in future longitudinal studies using structural equation modeling.

Despite the absence of a moderating effect, psychological safety remains an important characteristic of healthy clinical learning environments. The comparatively lower scores for help‐seeking behaviors suggest that some interns may still hesitate to seek assistance or acknowledge uncertainty during clinical practice. Encouraging open communication, constructive feedback, and supportive supervisory relationships therefore remains essential for promoting learning, preventing errors, and supporting the professional development of novice nurses [20, 34].

### 5.5. Sociodemographic Characteristics and Readiness for Practice

Beyond the influence of leadership, the adjusted regression analysis identified internship stage and university affiliation as independent predictors of readiness for practice. Interns in the middle stage of the internship reported lower readiness than those at the beginning of the program, suggesting that the transition from initial orientation to increasing clinical responsibility may represent a particularly demanding period. During this phase, interns are expected to assume greater autonomy while continuing to develop clinical competence and confidence, highlighting the need for sustained mentoring and leadership support throughout the internship rather than concentrating developmental support during orientation alone. This interpretation is supported by Gularte‐Rinaldo et al. [13], who demonstrated that sustained mentorship enhances novice nurses’ confidence and coping capacity, and by Jung and Kim [14], who reported that supportive mentorship facilitates professional identity formation during the transition to practice.

Institutional differences also emerged as significant predictors of readiness for practice. Although organizational characteristics were not examined directly, variation in clinical supervision, preceptor preparation, learning opportunities, and organizational culture may partially explain these findings. Similar observations were reported by AlThiga et al. [27], who emphasized the contribution of structured internship experiences to clinical preparedness, and by Almadani et al. [8], who highlighted the importance of supportive clinical learning environments in promoting readiness for professional practice. Future multicenter studies should explore organizational factors that may account for these institutional differences.

Although female nurse interns reported slightly higher readiness scores in the unadjusted analysis, gender was not independently associated with readiness after adjustment for demographic and educational characteristics. This finding indicates that educational experiences, leadership, and the quality of the clinical learning environment contribute more substantially to readiness for practice than demographic characteristics alone.

Overall, the findings indicate that readiness for practice is influenced primarily by leadership and the quality of the clinical learning environment rather than by demographic characteristics alone. Legacy leadership emerged as the strongest predictor of readiness for practice, underscoring the value of ethical role modeling, mentorship, and knowledge sharing in supporting interns’ transition to professional practice. These findings position legacy leadership as a promising framework for strengthening internship education and guiding nursing leaders in preparing a competent and confident future workforce.

### 5.6. Strengths and Limitations

This study extends the nursing leadership literature by providing one of the first empirical examinations of legacy leadership in relation to nurse interns’ readiness for practice. It introduces the researcher‐developed LLS and provides preliminary evidence supporting its application as a measurable leadership construct in nursing research. The findings also provide new insight into the role of psychological safety by demonstrating that it did not moderate the relationship between legacy leadership and readiness for practice, suggesting that alternative explanatory pathways warrant investigation. Finally, this multicenter study provides evidence from Saudi Arabia, addressing an important regional gap in the literature on transition‐to‐practice outcomes.

Despite these strengths, several limitations should be acknowledged. The cross‐sectional design precludes causal inference, and convenience sampling may limit the generalizability of the findings. Self‐reported measures collected at a single time point may also have introduced social desirability bias and common method variance. Although the LLS demonstrated satisfactory internal consistency and an acceptable preliminary factor structure, comprehensive psychometric validation has not yet been completed. The high internal consistency coefficient may also indicate overlap among some items. Consequently, the findings related to legacy leadership should be interpreted as preliminary until additional validation studies are conducted.

Future research should validate the LLS using independent samples, confirmatory factor analysis, and assessments of convergent, discriminant, and measurement invariance. Longitudinal studies using structural equation modeling are also needed to examine alternative pathways through which leadership and psychological safety influence readiness for practice.

### 5.7. Practical Implications

#### 5.7.1. For Nursing Practice and Management

The findings support leadership development as a strategic priority for healthcare organizations. Leadership development programs should strengthen mentoring, ethical leadership, constructive feedback, and collaborative practice among nurse preceptors and clinical supervisors. Structured mentorship, regular reflective debriefing, and supportive supervisory relationships may strengthen interns’ self‐regulation, professional confidence, and readiness for practice. Nursing managers should also promote clinical environments that encourage open communication and help‐seeking behaviors throughout the internship experience.

#### 5.7.2. For Nursing Education

Leadership development and structured mentorship should be introduced before and reinforced throughout internship training. Integrating reflective practice, guided mentorship, and leadership learning into undergraduate curricula may strengthen professional identity, clinical reasoning, and preparedness for professional practice. Early exposure to psychologically safe learning environments may also enhance students’ engagement and confidence during clinical training.

#### 5.7.3. For Future Research

Future studies should evaluate legacy leadership using longitudinal and multicenter designs and further validate the LLS across diverse nursing populations. Structural equation modeling may help clarify whether psychological safety functions as a mediator or through other indirect pathways linking leadership with readiness for practice. Qualitative and mixed‐methods studies may also provide a deeper understanding of how leadership behaviors shape interns’ transition into professional practice.

## 6. Conclusion

Legacy leadership emerged as a strong and independent predictor of readiness for practice among nurse interns, highlighting the importance of ethical role modeling, mentorship, and professional support during the transition from student to practicing nurse. Although psychological safety did not moderate this relationship, it remains an important characteristic of supportive clinical learning environments that facilitate learning and professional development. These findings position legacy leadership as a promising framework for strengthening internship education and guiding nursing leaders in preparing a competent, confident, and practice‐ready nursing workforce.

## Author Contributions

All authors contributed substantially to conception, design, data acquisition, analysis, interpretation, and manuscript preparation and revision.

## Funding

This research received no specific grant from any funding agency in the public, commercial, or not‐for‐profit sector.

## Disclosure

All authors read and approved the final manuscript.

## Ethics Statement

The study received approval from the Nursing Research Ethical Committee, King Abdulaziz University (NREC Ref. No. 2F.112). Electronic informed consent was obtained from all participants prior to data collection.

## Conflicts of Interest

The authors declare no conflicts of interest.

## Supporting Information

Additional supporting information can be found online in the Supporting Information section.

Supporting Materials. The following supporting files are provided with this manuscript:

## Supporting information


**Supporting Information 1** Supporting Table S1: Psychological safety scale item scores. Supporting Table S2: Psychometric summary of the Legacy Leadership Scale (KMO, Bartlett’s test, variance explained, and reliability). Supporting Table S3: Rotated factor loadings of the Legacy Leadership Scale. Supporting Table S4: Eigenvalues and variance explained of the Legacy Leadership Scale. Supporting Table S5: Reliability by domain of the Legacy Leadership Scale (Cronbach’s *α*).

**Supporting Information 2** STROBE checklist: the Strengthening the Reporting of Observational Studies in Epidemiology (STROBE) guidelines for cross‐sectional studies.

## Data Availability

Data are available on request from the authors. All included studies are publicly available through their respective publishers and are fully cited in the reference list.
