# Supplementary material for: Bridging the Gap: The Roles of Legacy Leadership and Psychological Safety in Nurse Interns’ Readiness for Practice
Source: J Nurs Manag. 2026 Aug 2;2026:4177127. doi: 10.1155/jonm/4177127 (PMC13429939; doi:10.1155/jonm/4177127)
Supplement: Supplementary file 1 — Supporting Information 1 Supporting Table S1: Psychological safety scale item scores. Supporting Table S2: Psychometric summary of the Legacy Leadership Scale (KMO, Bartlett’s test, variance explained, and reliability). Supporting Table S3: Rotated factor loadings of the Legacy Leadership Scale. Supporting Table S4: Eigenvalues and variance explained of the Legacy Leadership Scale. Supporting Table S5: Reliability by domain of the Legacy Leadership Scale (Cronbach’s α). [file JONM-2026-4177127-s001.docx]

**Supplementary Table S1**: Psychological safety scale item scores.

| **Item** | **M** | **SD** | **% of Max** |
| --- | --- | --- | --- |
| 1. Mistake held against you (R) | 5.23 | 1.80 | 74.7% |
| 2. Able to bring up problems | 5.56 | 1.50 | 79.5% |
| 3. Reject others for being different (R) | 4.92 | 1.99 | 70.3% |
| 4. Safe to take a risk | 5.38 | 1.52 | 76.9% |
| 5. Difficult to ask for help (R) | 4.39 | 2.25 | 62.7% |
| 6. No one undermines my efforts | 5.39 | 1.66 | 76.9% |
| 7. Skills valued and utilized | 5.93 | 1.26 | 84.8% |
| **Overall** | **37.55** | **9.47** | **76.6%** |

**Supplementary Table S2. Psychometric Summary of the Legacy Leadership Scale**

| **Measure** | **Result** |
| --- | --- |
| Number of items | 25 |
| Number of domains | 5 |
| KMO measure of sampling adequacy | 0.898 |
| Bartlett’s test of sphericity, χ² (df = 300) | 2596.31, p < .001 |
| Total variance explained (5-factor solution) | 80.70% |
| **Cronbach’s α (total scale)** | **0.977** |

*Note. KMO = Kaiser–Meyer–Olkin measure of sampling adequacy. Bartlett’s test of sphericity was significant, supporting factorability of the correlation matrix. Analyses were conducted on the subset of participants with complete item-level data (n = 89).*

**Supplementary Table S3. Rotated Factor Loadings of the Legacy Leadership Scale**

| **Item** | **Factor 1** | **Factor 2** | **Factor 3** | **Factor 4** | **Factor 5** |
| --- | --- | --- | --- | --- | --- |
| *Integrity & Ethical Leadership (Items 1–5)* | | | | | |
| Item 1 | -.03 | .08 | .06 | .53 | .38 |
| Item 2 | .11 | .03 | -.17 | .23 | .75 |
| Item 3 | -.26 | .33 | .42 | .45 | .10 |
| Item 4 | -.02 | .18 | .10 | -.04 | .77 |
| Item 5 | .24 | .27 | .20 | .53 | -.29 |
| *Mentoring & Empowering Others (Items 6–10)* | | | | | |
| Item 6 | .39 | .44 | -.22 | .28 | .05 |
| Item 7 | -.16 | .85 | .19 | -.02 | .07 |
| Item 8 | .21 | .69 | -.16 | .24 | -.02 |
| Item 9 | .27 | .59 | -.04 | -.01 | .16 |
| Item 10 | -.04 | .04 | .81 | .17 | -.04 |
| *Collaborative Leadership (Items 11–15)* | | | | | |
| Item 11 | .07 | -.07 | .82 | .30 | -.14 |
| Item 12 | .25 | -.05 | .00 | .67 | .08 |
| Item 13 | -.13 | .64 | .26 | .01 | .23 |
| Item 14 | .40 | .32 | .44 | -.24 | .10 |
| Item 15 | -.06 | -.03 | .20 | .82 | .08 |
| *Vision & Long-term Impact (Items 16–20)* | | | | | |
| Item 16 | .36 | .18 | .51 | -.18 | .17 |
| Item 17 | .48 | .30 | .02 | .24 | -.07 |
| Item 18 | .55 | .27 | .30 | -.06 | -.03 |
| Item 19 | .45 | .32 | .31 | .02 | -.13 |
| Item 20 | .59 | .18 | -.02 | .27 | -.02 |
| *Building Sustainable Systems (Items 21–25)* | | | | | |
| Item 21 | .49 | -.24 | .25 | .42 | .15 |
| Item 22 | .67 | .18 | .28 | -.06 | -.09 |
| Item 23 | .84 | -.29 | .33 | .00 | .05 |
| Item 24 | .69 | -.18 | .32 | -.04 | .21 |
| Item 25 | .88 | .07 | -.28 | .21 | .02 |

*Note. Principal axis factoring with oblique (promax) rotation; analyses based on the subset with complete item-level data (n = 89). Loadings ≥ .40 are considered salient* ***(shown in bold)*** *and indicate the items defining each factor.*

**Supplementary Table S4. Eigenvalues and Variance Explained of the Legacy Leadership Scale (**

| **Factor** | **Eigenvalue** | **Variance (%)** | **Cumulative (%)** |
| --- | --- | --- | --- |
| Factor 1 | 16.28 | 65.12 | 65.12 |
| Factor 2 | 1.28 | 5.11 | 70.23 |
| Factor 3 | 1.10 | 4.40 | 74.63 |
| Factor 4 | **0.78** | **3.12** | **77.75** |
| Factor 5 | **0.74** | **2.95** | 80.70 |

*Note. A theory-driven five-factor solution consistent with the conceptual framework was retained. Three factors exceeded an eigenvalue of 1.00. Factors 4 and 5 were retained on theoretical grounds despite eigenvalues below 1.00.*

**Supplementary Table S5. Reliability by Domain of the Legacy Leadership Scale**

| **Domain** | **No. of items** | **Cronbach’s α** |
| --- | --- | --- |
| Integrity and Ethical Leadership | 5 | 0.880 |
| Mentoring and Empowering Others | 5 | 0.898 |
| Collaborative Leadership | 5 | 0.892 |
| Vision and Long-Term Impact | 5 | 0.940 |
| Building Sustainable Systems | 5 | 0.931 |
| **Total Scale** | **25** | **0.977** |

*Note. Domain-level and total-scale reliability were computed on the subset of participants with complete item-level data (n = 89). The very high total-scale α is consistent with the full-sample estimate (α = 0.981) and may indicate item redundancy warranting future refinement.*
